# microRNA expression pattern as an ancillary prognostic signature for radiotherapy

**DOI:** 10.1186/s12967-018-1711-4

**Published:** 2018-12-05

**Authors:** An-Lun Li, Tao-Sang Chung, Yao-Ning Chan, Chien-Lung Chen, Shih-Chieh Lin, Yun-Ru Chiang, Chen-Huan Lin, Chi-Ching Chen, Nianhan Ma

**Affiliations:** 1 Department of Biomedical Sciences and Engineering, Institute of Systems Biology and Bioinformatics, National Central University, Taoyuan, Taiwan; 2 Department of Radiation Oncology, Landseed Hospital, Taoyuan, Taiwan; 3 Department of Nephrology, Landseed Hospital, Taoyuan, Taiwan; 4 Department of Pathology and Laboratory Medicine, Landseed Hospital, Taoyuan, Taiwan; 5 Institute of Basic Medical Sciences, College of Medicine, National Cheng Kung University, Tainan, Taiwan

**Keywords:** microRNAs, Radiotherapy, Biomarkers, Prognosis, Cancer

## Abstract

**Background:**

In view of the limited knowledge of plasma biomarkers relating to cancer resistance to radiotherapy, we have set up screening, training and testing stages to investigate the microRNAs (miRNAs) expression profile in plasma to predict between the poor responsive and responsive groups after 6 months of radiotherapy.

**Methods:**

Plasma was collected prior to and after radiotherapy, and the microRNA profiles were analyzed by quantitative reverse transcription polymerase chain reaction (qRT-PCR) arrays. Candidate miRNAs were validated by single qRT-PCR assays from the training and testing set. The classifier for ancillary prognosis was developed by multiple logistic regression analysis to correlate the ratios of miRNAs expression levels with clinical data.

**Results:**

We revealed that eight miRNAs expressions had significant changes after radiotherapy and the expression levels of miR-374a-5p, miR-342-5p and miR-519d-3p showed significant differences between the responsive and poor responsive groups in the pre-radiotherapy samples. The Kaplan–Meier curve analysis also showed that low miR-342-5p and miR-519d-3p expressions were associated with worse prognosis. Our results revealed two miRNA classifiers from the pre- and post-radiotherapy samples to predict radiotherapy response with area under curve values of 0.8923 and 0.9405.

**Conclusions:**

The expression levels of miR-374a-5p, miR-342-5p and miR-519d-3p in plasma are associated with radiotherapy responses. Two miRNA classifiers could be developed as a potential non-invasive ancillary tool for predicting patient response to radiotherapy.

**Electronic supplementary material:**

The online version of this article (10.1186/s12967-018-1711-4) contains supplementary material, which is available to authorized users.

## Background

With more than half of the cancer patients will receive radiation therapy as part of treatment in head and neck and rectal cancer, recurrence is still a major cause of treatment failure despite the advances of combination chemo-irradiation and preoperative radiotherapy [1]. Hence many studies have investigated the tumor radioresistance and signaling pathway [2–4]. Interestingly, it has been reported that the IGF1R, MAPK, PI3K and DNA repair signaling pathways are associated with radioresistance in several cancers [5–8]. However, the lack of a sensitive biomarker for radiotherapy and an understanding of mechanisms of related radioresistance hinder the success of radiation as a treatment for many patients [9–12].

The circulating miRNA profile is believed to be a molecular tool as disease biomarkers to predict or differentiate different types of disease [13–15]. The expression level of circulating miRNAs was related to the progression and development of cancers [13, 16]. In addition, circulating miRNA can be packaged in exosomes, the microvasculature or innate structures, enhancing its stability and avoiding degradation in biofluids [17, 18].

A large number of studies have examined the general and specific effects of miRNA perturbation in radiation-exposed cells [19–25]. However, cell line-based studies do not always correlate well with the results from clinical studies and no reliable and predictive biomarker could be applied in clinical for radiotherapy. Therefore, investigations on the non-invasive way to assess miRNA expression patterns to predict radiotherapy response are our primary interest. In the present work, we aimed to study the effects of radiotherapy on the expression levels of miRNAs in plasma. We further used these miRNA signatures to develop prediction classifiers for samples with an unknown radiotherapy status.

## Materials and methods

### Patients and samples

A total of 62 patients, including 26 and 36 patients with non-metastatic rectal cancer and head and neck cancers, respectively, were enrolled from December 2012–2015 (LSH-IRB-12-15). All patients were treated with radiation as part of curative treatment using a linear accelerator (6 MV, 10 MV) with standard dose fraction (2 Gy per day). Treatment response was evaluated 3 to 6 months after treatment including computed tomography (CT) imaging, magnetic resonance (MR) imaging and positron emission tomography (PET) imaging. Primary tumor with complete and partial response was defined as responsive group and the other as poor responsive. Response assessed with the use of response evaluation criteria in solid tumors (RECIST), version 1.1 [26]. In all, 15 poor responsive and 47 responsive patients were compared in this study.

Peripheral blood samples were collected from patients after obtaining informed consent. The samples were collected within 5 days before and after conclusion of radiotherapy. Samples were centrifuged and separated into plasma and carefully stored at − 80 °C.

### RNA isolation from plasma samples

Total RNA from 0.5 ml of plasma was extracted by using TRIzol^®^ LS Reagent and a mirVana™ miRNA Isolation Kit according to the standard protocol. We used Syn-cel-miR-39 as spiked-in control for some of the technical variability of plasma RNA extraction. The median of the syn-cel-miR-39 CT value obtained from all the samples was calculated. The RNA quality from the plasma was detected by a spectrophotometer (BioTek). All the RNA samples were carefully stored at − 80 °C.

### Reverse transcription

cDNAs were reverse transcribed from miRNAs using a TaqMan™ MicroRNA Reverse Transcription Kit (Applied Biosystems) with 600 ng of total RNA and miRNA specific stem loop primers including miRNA PCR array A (Megaplex RT primers for Human Pool A) and the miRNA candidate pool. The conditions for reverse transcription were in accordance with the standard protocol. cDNA was generated using TaqMan^®^ 2× Universal PCR master mix without UNG and TaqMan^®^ Array Human MicroRNA Cards A or TaqMan^®^ miRNA single assays.

### MiRNA profiling and individual miRNA quantification by RT-PCR

miRNA PCR profiling in plasma samples were carried out using TaqMan^®^ Array Human MicroRNA Cards (Applied Biosystems). To quantify individual miRNA levels, we used TaqMan^®^ miRNA single assays as the main detection method as described before [27]. The expression of miRNAs was determined using the 2^−ΔCT^ method relative to U6. The raw data of miRNA expressions was transformed to log10 form since the data with log10 form was in accordance with the normal distribution. In our analysis, the value of no detection miRNAs expression was replaced into − 4.5 value at log10 form.

### Survival curve analysis

A public website of a smRNA-seq analysis of the clinical specimens was compared to survival status at YM500v3: a database for small RNA sequencing in human cancer research (http://driverdb.tms.cmu.edu.tw/ym500v3/index.php) [28]. Then, miR-374a-5p, miR-342-5p and miR-519d-3p expression values from clinical specimens were used to perform Kaplan–Meier survival curve analysis according to the clinical parameter provided in the same dataset. High and low expression groups were created by using the quantile and median value, respectively as a cutoff.

### Data statistical analysis

Clinical characteristics between poor responsive and responsive patients were evaluated by using Pearson’s Chi squared test for categorical variables. Normality and Student’s *t* test were used for unpaired comparisons of two groups. All tests were two-tailed and were assessed by Levene’s test. All the statistical analyses were completed with GraphPad Prism software. The logistic regression of miRNA ratios combination were completed with SigmaPlot software.

## Results

### Identification of differentially expressed candidate miRNAs in plasma between poor responsive and responsive groups of radiotherapy

To identify potential miRNA signatures as a prognostic tool for radiotherapy patients, the miRNA profiles of plasma screened by high-throughput real-time miRNA PCR array were first reviewed. The plasma from patients was collected in prior to radiotherapy and after completion of radiotherapy (Additional file 1: Fig. S1). We monitored each patient’s condition at 6 months after radiotherapy, and the patients were characterized as poor responsive or responsive according to RECIST criteria by medical doctors. The screening set includes eight plasma samples collected prior to radiotherapy and seven plasma samples collected after radiotherapy (Table 1). The miRNA expression profiles in the plasma from poor responsive and responsive patients were compared. Total 22 candidate miRNAs were selected from the screening results (Additional file 1: Table S1).

**Table 1:** Distribution of the clinical status of patients in this study

	Before radiation													
	Screening set (n = 8)							Training set (n = 38)						
	Poor response			Response			P	Poor response			Response			P
	N	Mean	SD	N	Mean	SD		N	Mean	SD	N	Mean	SD	
Type^b^														
H&N	2			2			1	8			17			0.69
Colorectal	2			2				5			8			
Age^a^	4	69.75	16.24	4	62	16.81	0.52	13	61.54	15.03	25	61.08	15.21	0.93
Sex^b^														
Female	1			1			1	6			13			0.732
Male	3			3				7			12			
Stage^b^														
I	1			1			0.23	1			4			0.111
II	0			2				0			6			
III	3			1				4			8			
IV	0			0				8			7			
Total dosage^a^ (Gy)	3	63.87	11.68	4	60.25	11.37	0.69	13	66.18	7.58	24	63.61	9.29	0.398
Chemotherapy^b^	4			4			1	13			25			0.728

	Before radiation						
	Testing (n = 24)						
	Poor response			Response			P
	N	Mean	SD	N	Mean	SD	
Type^b^							
H&N	1			10			0.902
Colorectal	1			12			
Age^a^	2	69.5	9.19	22	54.45	11.99	0.1
Sex^b^							
Female	2			15			0.343
Male	0			7			
Stage^b^							
I	0			3			0.081
II	0			6			
III	0			8			
IV	2			5			
Total dosage^a^ (Gy)	2	60.2	13.86	22	58.03	14.7	0.843
Chemotherapy^b^	2			22			0.577

	After radiation													
	Screening set (n = 7)							Training set (n = 31)						
	Poor response			Response			P	Poor response			Response			P
	N	Mean	SD	N	Mean	SD		N	Mean	SD	N	Mean	SD	
Type^b^														
H&N	2			2			0.66	7			15			0.94
Colorectal	1			2				3			6			
Age^a^	3	62.67	9.71	4	62	16.81	0.95	10	58.6	13.23	21	60.29	14.73	0.75
Sex^b^														
Female	1			1			0.81	5			11			0.9
Male	2			3				5			10			
Stage^b^														
I	0			1			0.14	0			3			0.042*
II	0			2				0			5			
III	3			1				2			7			
IV	0			0				8			6			
Total dosage^a^ (Gy)	2	70.6	0.85	4	60.25	11.37	0.29	10	66.88	6.63	20	64.28	9.01	0.43
Chemotherapy^b^	3			4			0.81	10			21			0.36

	After radiation						
	Testing (n = 24)						
	Poor response			Response			P
	N	Mean	SD	N	Mean	SD	
Type^b^							
H&N	1			10			0.902
Colorectal	1			12			
Age^a^	2	69.5	9.19	22	54.45	11.99	0.1
Sex^b^							
Female	2			15			0.343
Male	0			7			
Stage^b^							
I	0			3			0.081
II	0			6			
III	0			8			
IV	2			5			
Total dosage^a^ (Gy)	2	60.2	13.86	22	58.03	14.7	0.843
Chemotherapy^b^	2			22			0.577

Each groups were well matched for age, gender. Mean, average of each samples

*SD* standard deviation, *M* male, *F* female

*P value < 0.05

^a^Independent samples test

^b^Pearson Chi Square

### Changes in the miRNA expression levels after radiotherapy

**Fig. 1:**
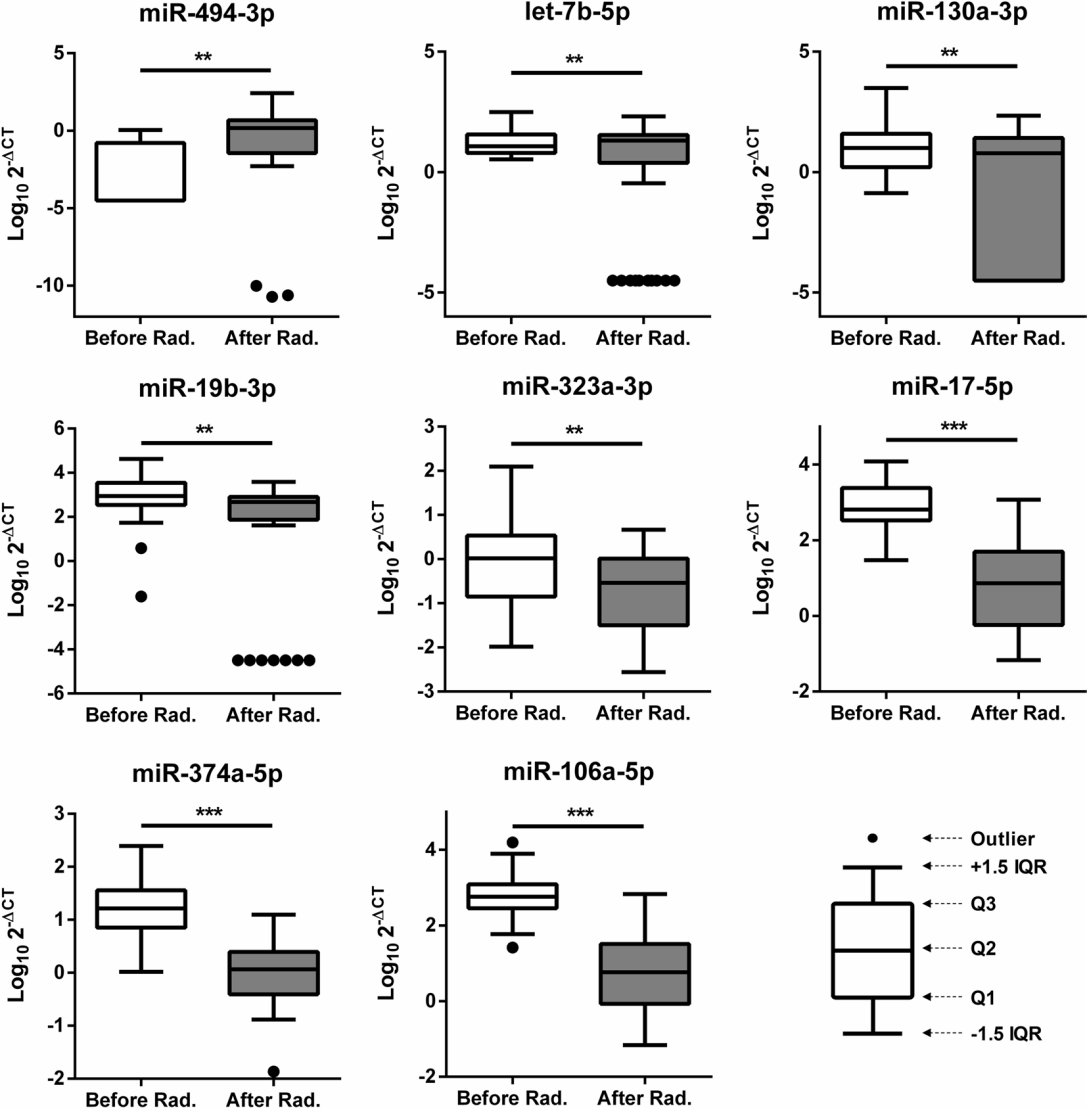
Significant changes in the miRNAs expression levels in plasma after radiotherapy. miRNA levels from the plasma of patients detected by qRT-PCR using RNU6 as a control. The Y axis presents the expression level (Log_10_^2−ΔCT^). *Rad* radiation. Student’s t-test: *P value < 0.05; **P value < 0.01; ***P value < 0.001

Investigations on the possible influence of radiotherapy on miRNA expression patterns were of primary interest. To validate the data from our screen, we checked the expression of 22 candidate miRNAs from the training set by single qRT-PCR. The training set included 38 different plasma samples collected from patients prior to radiotherapy and 31 different plasma samples collected from patients after radiotherapy (Table 1). First, we examined whether radiotherapy exerted any changes in these candidate miRNA expressions. Our results showed that eight miRNAs had significantly different expression levels after radiotherapy. miR-494-3p and let-7b-5p expression was increased, but the other six miRNAs—miR-130a-3p, miR-19b-3p, miR-323a-3p, miR-17-5p, miR-374a-5p, and miR-106a-5p—had significantly decreased expression (Fig. 1). We also assessed miRNA changes among the same patients before and after radiotherapy by the paired t-test. Nine miRNAs expressions including miR-299-5p and eight miRNAs above showed significant difference before and after radiotherapy (Additional file 1: Fig. S2). Interestingly, radiation-triggered deregulation of miR-494-3p, let-7b-5p, and miR-106a-5p has also been reported in previous studies [5, 13, 24, 25, 29–32].

### miRNAs expression levels linked to radiotherapy responses

**Fig. 2:**
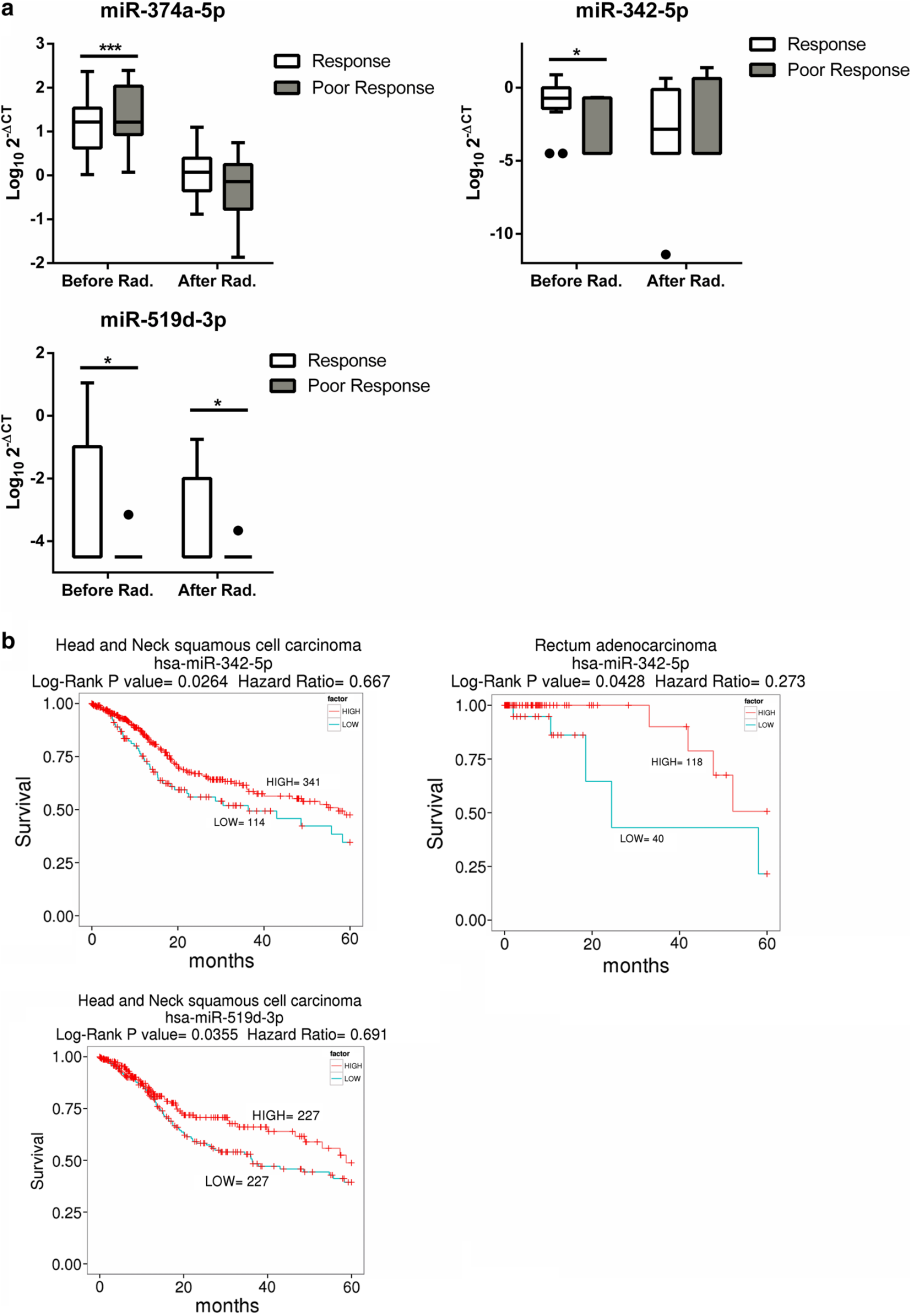
The difference in the miRNA expression levels between the poor responsive and responsive groups. **a** Difference in the miRNA expression in the poor responsive vs responsive groups before or after radiation. miRNA levels from the plasma of patients detected by qRT-PCR using RNU6 as a control. **b** The Kaplan–Meier survival curve of head and neck patients: low miRNA expression versus high miRNA expression. The statistical significance of the difference between the two groups was showed. The Y axis presents the expression level (Log_10_^2−ΔCT^). *Rad* radiation. Student’s t-test: *P value < 0.05; **P value < 0.01; ***P value < 0.001

We further analyzed whether the plasma miRNA expression levels were associated with prognostic responses 6 months after radiotherapy. We investigated which candidate miRNA expression levels were different between the poor responsive and responsive groups (Fig. 2a). High expression levels of miR-374a-5p and low expression levels of miR-342-5p and miR-519d-3p prior to radiotherapy were observed in the poor responsive group (p < 0.0001, p = 0.044 and p = 0.014, respectively). In addition, low expression levels of miR-519d-3p after radiation were also shown in the poor responsive group (p = 0.0251). These results suggest that higher levels of miR-374a-5p and lower levels of miR-342-5p or miR-519d-3p in plasma could be linked to worse prognosis. Interestingly, the previous study reported that miR-374b-5p expression is linked to the radiation resistance in HNSCC [13]. Further, we utilized a public website of smRNA-seq analysis of the clinical cancer specimens [28]. Kaplan–Meier plot was analyzed to check for an association between miR-374a-5p, miR-342-5p or miR-519d-3p expression and 5-year survival. (Fig. 2b and Additional file 1: Fig. S3). Interestingly, both of head and neck squamous cell carcinoma and rectum adenocarcinoma patients with low miR-342-5p expression had significantly shorter survival than those in higher expression group (p = 0.0264 and 0.0428, respectively). Lower miR-519d-3p expression also had significantly shorter 5-year survival (p = 0.0355).

To develop a miRNA signature-based predicative model for patients with unknown radiation responses, we carried out a ROC analysis for the all candidate miRNAs (Additional file 1: Table S2). The AUC values of let-7b-5p and miR-342-5p were 0.722 and 0. 762, respectively, in the pre-radiotherapy samples (Table 2A). These values suggest that plasma let-7b-5p and miR-342-5p levels are good potential candidates for radiotherapy biomarkers.

**Table 2:** The discriminatory ability of the miRNA expression profile for the poor responsive and responsive groups

A		
miRNA	AUC	
	Before radiation	After radiation
miR-494-3p	0.552	0.636
let-7b-5p	0.722	0.552
miR-323a-3p	0.562	0.512
miR-19b-3p	0.603	0.547
miR-342-5p	0.762	0.527
miR-374a-5p	0.568	0.625
miR-519d	0.658	0.647

B	
miRNA ratio	AUC
Before radiation	
130a-3p/let-7b-5p	0.788
130a-3p/19b-3p	0.763
130a-3p/374a-5p	0.763
130a-3p/17-5p	0.745
106a-5p/130a-3p	0.732
After radiation	
130a-3p/let-7b-5p	0.752
628-5p/let-7b-5p	0.714
130a-3p/148a-3p	0.686
148a-3p/494-3p	0.676
148a-3p/628-5p	0.671

The expression of 22 candidate miRNAs were changed to the ratio form to eliminate normalization issue in plasma. The top five miRNA ratios were statistically calculated their AUC values by ROC analysis form (A) before radiation group and (B) after radiation group

### Set up two classifiers to predict radiotherapy responses

**Fig. 3:**
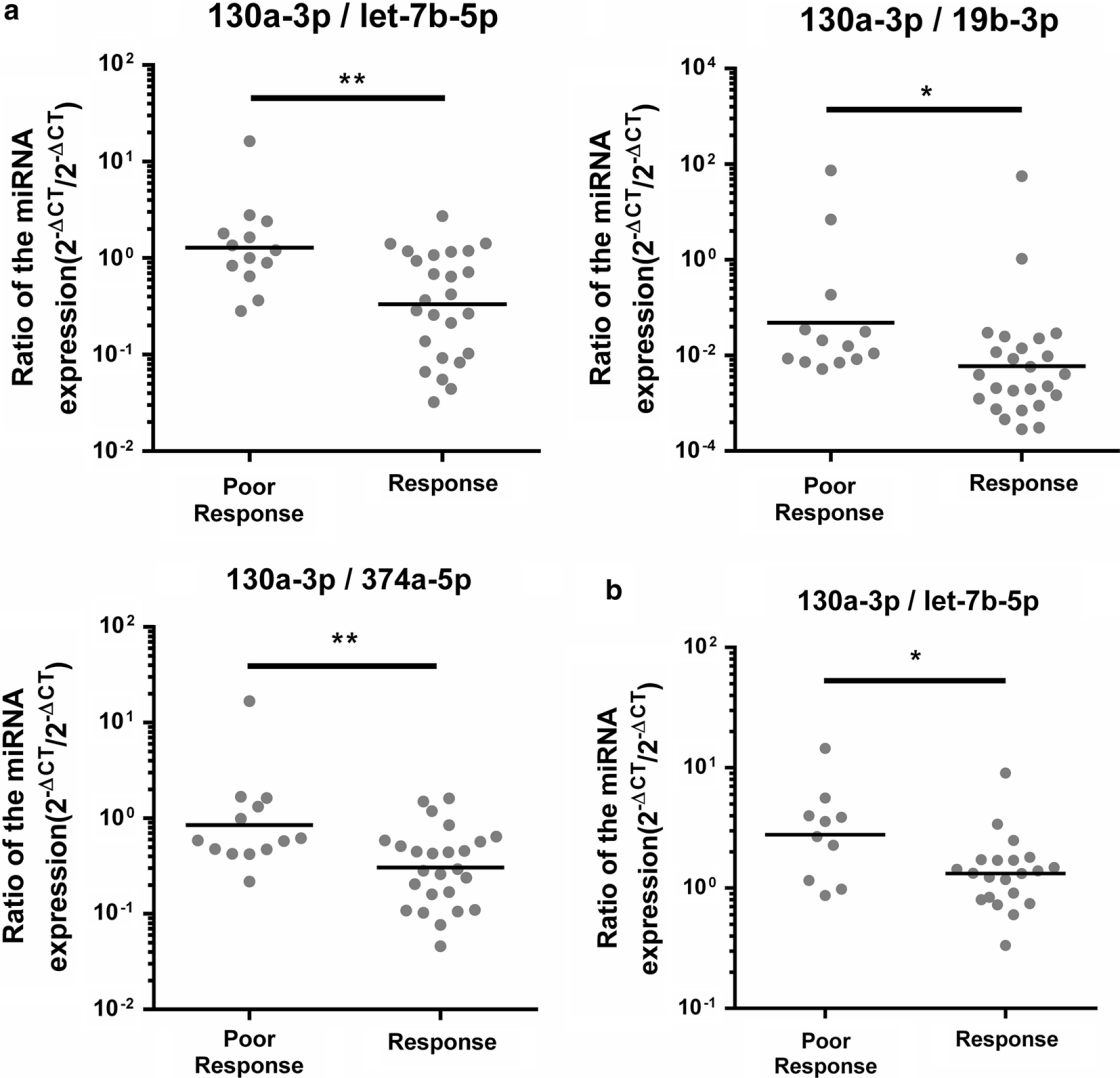
The scatter plots of miRNAs expression ratio. **a** The scatter plots of miR-130a-3p/let-7b-5p, miR-130a-3p/miR-19b-3p and miR-130a-3p/miR-374a-5p were shown to distinguish responsive or poor responsive in the pre-radiation samples. **b** The scatter plots of miR-130a-3p/let-7b-5p was shown to distinguish responsive or poor responsive in the post-radiation samples. The Y axis was presents the ratio (2^−ΔCT^/2^−ΔCT^). Student’s t-test: *P value < 0.05; **P value < 0.01; ***P value < 0.001

There is no strong evidence to indicate which miRNA or non-coding RNA is the appropriate internal control to normalize miRNA expression levels in plasma so far. Therefore, we utilized the ratio method, which divided two miRNAs expression levels from the same sample to eliminate the normalization issue. We calculated all miRNAs combination ratios and selected the miRNAs combination ratios with top five values of AUC (Table 2B). In the pre-radiation samples, the ratio levels of miR-130a-3p/let-7b-5p, miR-130a-3p/miR-19b-3p, and miR-130a-3p/miR-374a-5p were significantly different between poor responsive and responsive patients (p = 0.00122, 0.0419, and 0.0087, respectively) (Fig. 3a), and their AUC values were 0.788, 0.763, and 0.763, respectively. Interestingly, in the post-radiotherapy samples, the ratio levels of miR-130a-3p/let-7b-5p was also significantly different (p = 0.03147) (Fig. 3b), and its AUC value to discriminate poor responsive from responsive patients was 0.752.

**Fig. 4:**
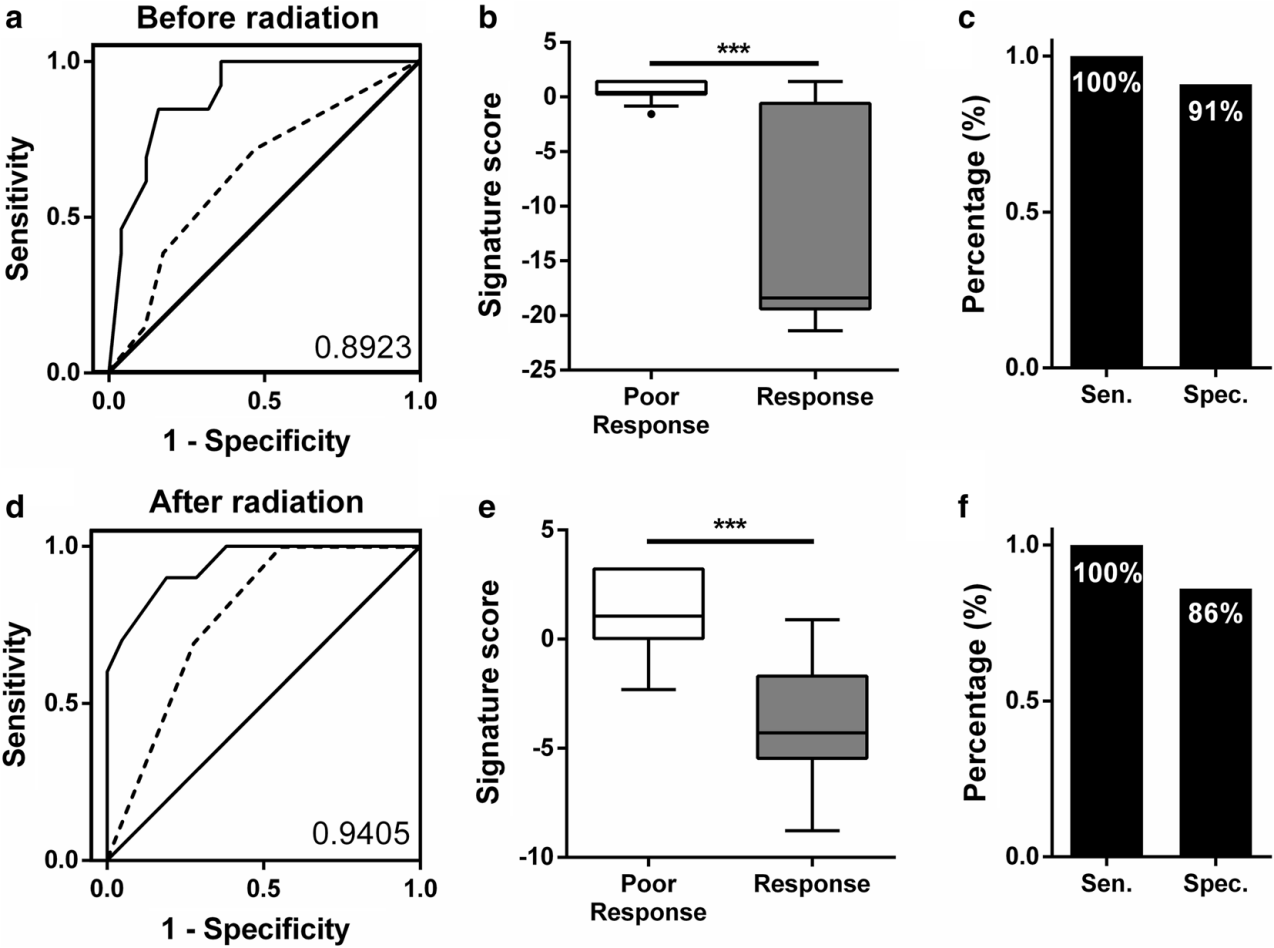
The ROC analysis of miRNA combinations. **a**–**c** The ROC analysis for miR-130a-3p/let-7b-5p, miR-130a-3p/miR-19b-3p, miR-130a-3p/miR-374a-5p and tumor stage were shown to distinguish responsive or poor response patients before radiation treatment. **d**–**f** The ROC analysis for the miR-130a-3p/let-7b-5p, miR-130a-3p/miR-148a-3p and tumor stage were shown to distinguish responsive or poor responsive patients after radiation treatment. Dashed line presents the ROC of tumor stage. **b**, **e** The box plots show the classifier distribution that combines the miRNA ratios with clinical data from training set. **c**, **f** The histogram shows the best accuracy of the sensitivity and specificity from the testing set. *Res* responsive patients, *Poor* poor responsive patients, *Sen* sensitivity, *Spec* specificity, *AUC* area under the curve

To establish a proper model to further estimate the radiotherapy responses, the different ratios of the miRNA data from the training set were combined to calculate the formula using multiple logistic regression. Therefore, we established two classifiers that could significantly distinguish the poor responsive from responsive patients, and these two classifiers could predict the radiation responses 6 months after radiotherapy (Fig. 4). For the pre-radiotherapy samples (n = 38), the classifier including three miRNA ratios—miR-130a-3p/let-7b-5p, miR-130a-3p/miR-19b-3p and miR-130a-3p/miR-374a-5p—with tumor stage data, and the AUC values was 0.8923 (95% CI 0.7910 to 0.9936) (Fig. 4a). For post-radiotherapy samples (n = 31), the AUC of the classifier, which included two miRNA ratios—miR-130a-3p/let-7b-5p and miR-130a-3p/miR-148a-3p—with tumor stage data, reached 0.9405 (95% CI 0.8591 to 1.022) (Fig. 4d). We further analyzed the distribution of the two signatures (Fig. 4b, e). Moreover, we validated these two signatures by testing another sample set (n = 24). In the pre-radiation samples, the classifier could detect poor responsive from responsive patients at a cut-off point of 0.2145 with 100% sensitivity and 91% specificity (Fig. 4c). In the post-radiation samples, the classifier could detect poor responsive from responsive patients at a cut-off point of 2.865 with 100% sensitivity and 86% specificity (Fig. 4f). Poor responsive patients were identified by both classifiers.

## Discussion

In this study, we aimed to establish plasma miRNAs as ancillary predictive biomarkers for radiotherapy. Furthermore, we compared miRNA expression before and after treatment, and revealed that the expression levels of eight miRNAs had significant changes after radiotherapy. Interestingly, in the pre-radiation samples, we revealed that the expression levels of miRNA-374-5p, miR-342-5p and miR-519d-3p were significantly different between the responsive and poor responsive groups. These data suggested that the expression levels of three miRNAs may influence radiation sensitivity.

The let-7 family of miRNAs is a group of well-known tumor suppressor miRNAs, and many studies showed its levels are affected by radiation in vitro and in vivo [20]. Among them, let-7b is transcriptionally repressed by p53, and this mechanism depends on functional p53 and radiation-activated ATM signaling [33]. In mice with functional p53, a decrease in let-7b levels was observed in the more radiosensitive tissues upon radiation. These results are consistent with our finding that the let-7b-5p levels significantly decreased only in the plasma of the radiotherapy responsive group. Previous studies showed that the levels of miR-494-3p increased upon radiation in glioma cells [30]. Moreover, miR-494-3p could induce the radiosensitivity of oral squamous cell carcinoma by downregulating Bmi1 [25]. We similarly observed that the levels of miR-494-3p are increased after radiotherapy, and higher levels of miR-494-3p were expressed in the responsive group. Furthermore, it has also been reported that levels of miR-19b and miR-17 decreased in lymphocytes after radiation [20, 31]. However, changes in the miR-106 levels were observed in lung, thyroid MCF-7 and blood cells after radiation [20, 21, 34, 35]. In addition, the decrease or increase in these miRNA levels may not be consistent between cells and plasma, which may be due to tissue-specific or functional differences between cells and extracellular conditions.

Our results showed that three initial miRNAs in plasma—miR-374a-5p, miR-342-5p and miR-519d-3p—are involved in the prognosis of radiation responses as shown in Fig. 2. Summerer et al. demonstrated that high expression of miR-374b-5p in the plasma of individuals with HNSCC correlated with worse prognosis. Interestingly, miR-374a-5p and miR-374b-5p are present in the same seed region, so both of them may regulate the same radiation response-related genes. However, the mechanisms of miR-374a-5p and the other two miRNAs, miR-342-5p and miR-519d-3p, involved in radiotherapy responses were unclear until now, and our results show that three miRNAs have low AUC values for predicting radiotherapy outcomes. In addition, previous studies showed the expression of miR-296-5p and miR-16 have changed after radiotherapy and proposed that their expressions were related to the patients’ survival [10, 36]. However, small sample size or lack of sufficient predictive model to assess prognosis of radiotherapy in these studies limited the application in clinical use.

We applied each candidate miRNAs expression level to the combination of the ratio of miRNAs expression and tumor stage data, which produced two classifiers to predict radiotherapy outcomes 6 months after radiotherapy. The combination of the expression ratios levels of miR-130a-3p/let-7b-5p, miR-130a-3p/miR-19b-3p, and miR-130a-3p/miR-374a-5p and the tumor stage were up-regulated in poor responsive patients’ pre-radiotherapy samples. Moreover, the combination of the expression ratios of miR-130a-3p/let-7b-5p and miR-130a-3p/miR-148a-3p were up-regulated in poor responsive patients’ post-radiotherapy samples. It is noted that both classifiers contained miR-130 expression. High miR-130 expression has been found in radiation-resistant lung and prostate cells [5, 37]. We observed that miR-130 expression levels was significantly decreased in plasma but no significant differences were observed between the poor responsive and responsive groups after radiation. Therefore, we established two miRNA bio-signature models that could act as ancillary prognostic tools for radiotherapy patients, to predict responses 6 months after radiotherapy, which revealed 100% sensitivity in the testing set. If poor responsive can be identified before or just after initial radiotherapy, the patient may receive an alternative radiation process or other active therapy. However, any bio-signature requires multiple cohorts to validate its reproducibility, and then it can be applied as a clinical biomarker. The two classifiers in this study to predict radiotherapy outcomes require more validation in different cohorts and different types of cancer.

## Conclusions

To date, no clinical tools could predict the therapeutic effects of radiation therapy. This study applied the miRNAs expression in plasma as ancillary predictive biomarkers for prognosis of radiotherapy. The expressions of miR-374a-5p, miR-342-5p and miR-519d-3p were observed the significant difference between the radiotherapy outcomes in prior of radiotherapy. Patients with lower miR-342-5p or miR-519d-3p expression had significantly shorter 5-year survival. Two classifiers were established from pre- and post-radiotherapy samples to predict radiotherapy outcome 6 months after radiotherapy with area under the curve (AUC) values of 0.8923 and 0.9405.

## Additional file

**Additional file 1.** Additional figures and tables.

## Abbreviations

miRNA: microRNA
AUC: area under the curve
CT: computed tomography
MRI: magnetic resonance imaging
PET: positron emission tomography
RECIST: response evaluation criteria in solid tumors
ROC: receiver operating characteristic
qRT-PCR: quantitative reverse transcription polymerase chain reaction

## References

[1] Delaney G, Jacob S, Featherstone C, Barton M (2005). The role of radiotherapy in cancer treatment: estimating optimal utilization from a review of evidence-based clinical guidelines. Cancer.

[2] Baumann M, Krause M, Hill R (2008). Exploring the role of cancer stem cells in radioresistance. Nat Rev Cancer.

[3] Toustrup K, Sorensen BS, Metwally MA, Tramm T, Mortensen LS, Overgaard J, Alsner J (2016). Validation of a 15-gene hypoxia classifier in head and neck cancer for prospective use in clinical trials. Acta Oncol.

[4] Ishigami T, Uzawa K, Higo M, Nomura H, Saito K, Kato Y, Nakashima D, Shiiba M, Bukawa H, Yokoe H (2007). Genes and molecular pathways related to radioresistance of oral squamous cell carcinoma cells. Int J Cancer.

[5] Wang XC, Du LQ, Tian LL, Wu HL, Jiang XY, Zhang H, Li DG, Wang YY, Wu HY, She Y (2011). Expression and function of miRNA in postoperative radiotherapy sensitive and resistant patients of non-small cell lung cancer. Lung Cancer.

[6] Salim H, Akbar NS, Zong D, Vaculova AH, Lewensohn R, Moshfegh A, Viktorsson K, Zhivotovsky B (2012). miRNA-214 modulates radiotherapy response of non-small cell lung cancer cells through regulation of p38MAPK, apoptosis and senescence. Br J Cancer.

[7] Qu JQ, Yi HM, Ye X, Zhu JF, Yi H, Li LN, Xiao T, Yuan L, Li JY, Wang YY (2015). MiRNA-203 reduces nasopharyngeal carcinoma radioresistance by targeting IL8/AKT Signaling. Mol Cancer Ther.

[8] Tommelein J, De Vlieghere E, Verset L, Melsens E, Leenders J, Descamps B, Debucquoy A, Vanhove C, Pauwels P, Gespach CP (2018). Radiotherapy-activated cancer-associated fibroblasts promote tumor progression through paracrine IGF1R activation. Cancer Res.

[9] Jacob NK, Cooley JV, Yee TN, Jacob J, Alder H, Wickramasinghe P, Maclean KH, Chakravarti A (2013). Identification of sensitive serum microRNA biomarkers for radiation biodosimetry. PLoS ONE.

[10] Yu Q, Li B, Li P, Shi Z, Vaughn A, Zhu L, Fu S (2015). Plasma microRNAs to predict the response of radiotherapy in esophageal squamous cell carcinoma patients. Am J Transl Res.

[11] Moertl S, Mutschelknaus L, Heider T, Atkinson MJ (2016). MicroRNAs as novel elements in personalized radiotherapy. Transl Cancer Res.

[12] Kerns SL, Dorling L, Fachal L, Bentzen S, Pharoah PD, Barnes DR, Gomez-Caamano A, Carballo AM, Dearnaley DP, Peleteiro P (2016). Meta-analysis of genome wide association studies identifies genetic markers of late toxicity following radiotherapy for prostate cancer. EBioMedicine.

[13] Summerer I, Unger K, Braselmann H, Schuettrumpf L, Maihoefer C, Baumeister P, Kirchner T, Niyazi M, Sage E, Specht HM (2015). Circulating microRNAs as prognostic therapy biomarkers in head and neck cancer patients. Br J Cancer.

[14] Lu J, Getz G, Miska EA, Alvarez-Saavedra E, Lamb J, Peck D, Sweet-Cordero A, Ebert BL, Mak RH, Ferrando AA (2005). MicroRNA expression profiles classify human cancers. Nature.

[15] Schena FP, Serino G, Sallustio F (2014). MicroRNAs in kidney diseases: new promising biomarkers for diagnosis and monitoring. Nephrol Dial Transplant.

[16] Kosaka N, Iguchi H, Ochiya T (2010). Circulating microRNA in body fluid: a new potential biomarker for cancer diagnosis and prognosis. Cancer Sci.

[17] Mall C, Rocke DM, Durbin-Johnson B, Weiss RH (2013). Stability of miRNA in human urine supports its biomarker potential. Biomark Med.

[18] Mitchell PS, Parkin RK, Kroh EM, Fritz BR, Wyman SK, Pogosova-Agadjanyan EL, Peterson A, Noteboom J, O’Briant KC, Allen A (2008). Circulating microRNAs as stable blood-based markers for cancer detection. Proc Natl Acad Sci USA.

[19] Gandellini P, Rancati T, Valdagni R, Zaffaroni N (2014). miRNAs in tumor radiation response: bystanders or participants?. Trends Mol Med.

[20] Metheetrairut C, Slack FJ (2013). MicroRNAs in the ionizing radiation response and in radiotherapy. Curr Opin Genet Dev.

[21] Templin T, Paul S, Amundson SA, Young EF, Barker CA, Wolden SL, Smilenov LB (2011). Radiation-induced micro-RNA expression changes in peripheral blood cells of radiotherapy patients. Int J Radiat Oncol Biol Phys.

[22] Xu S, Wang J, Ding N, Hu W, Zhang X, Wang B, Hua J, Wei W, Zhu Q (2015). Exosome-mediated microRNA transfer plays a role in radiation-induced bystander effect. RNA Biol.

[23] Wu SY, Wu AT, Liu SH (2016). MicroRNA-17-5p regulated apoptosis-related protein expression and radiosensitivity in oral squamous cell carcinoma caused by betel nut chewing. Oncotarget.

[24] Huang T, Yin L, Wu J, Gu JJ, Wu JZ, Chen D, Yu HL, Ding K, Zhang N, Du MY (2016). MicroRNA-19b-3p regulates nasopharyngeal carcinoma radiosensitivity by targeting TNFAIP3/NF-kappaB axis. J Exp Clin Cancer Res.

[25] Weng JH, Yu CC, Lee YC, Lin CW, Chang WW, Kuo YL (2016). miR-494-3p induces cellular senescence and enhances radiosensitivity in human oral squamous carcinoma cells. Int J Mol Sci.

[26] Eisenhauer EA, Therasse P, Bogaerts J, Schwartz LH, Sargent D, Ford R, Dancey J, Arbuck S, Gwyther S, Mooney M (2009). New response evaluation criteria in solid tumours: revised RECIST guideline (version 1.1). Eur J Cancer.

[27] Liu SM, Lu J, Lee HC, Chung FH, Ma N (2014). miR-524-5p suppresses the growth of oncogenic BRAF melanoma by targeting BRAF and ERK2. Oncotarget.

[28] Chung IF, Chang SJ, Chen CY, Liu SH, Li CY, Chan CH, Shih CC, Cheng WC (2017). YM500v3: a database for small RNA sequencing in human cancer research. Nucleic Acids Res.

[29] Marta GN, Garicochea B, Carvalho AL, Real JM, Kowalski LP (1992). MicroRNAs, cancer and ionizing radiation: where are we?. Rev Assoc Med Bras.

[30] Kwak SY, Yang JS, Kim BY, Bae IH, Han YH (2014). Ionizing radiation-inducible miR-494 promotes glioma cell invasion through EGFR stabilization by targeting p190B rhoGAP. Biochim Biophys Acta.

[31] Girardi C, De Pitta C, Casara S, Sales G, Lanfranchi G, Celotti L, Mognato M (2012). Analysis of miRNA and mRNA expression profiles highlights alterations in ionizing radiation response of human lymphocytes under modeled microgravity. PLoS ONE.

[32] Hummel R, Hussey DJ, Haier J (2010). MicroRNAs: predictors and modifiers of chemo- and radiotherapy in different tumour types. Eur J Cancer.

[33] Saleh AD, Savage JE, Cao L, Soule BP, Ly D, DeGraff W, Harris CC, Mitchell JB, Simone NL (2011). Cellular stress induced alterations in microRNA let-7a and let-7b expression are dependent on p53. PLoS ONE.

[34] Abou-El-Ardat K, Monsieurs P, Anastasov N, Atkinson M, Derradji H, De Meyer T, Bekaert S, Van Criekinge W, Baatout S (2012). Low dose irradiation of thyroid cells reveals a unique transcriptomic and epigenetic signature in RET/PTC-positive cells. Mutat Res.

[35] Shin S, Cha HJ, Lee EM, Lee SJ, Seo SK, Jin HO, Park IC, Jin YW, An S (2009). Alteration of miRNA profiles by ionizing radiation in A549 human non-small cell lung cancer cells. Int J Oncol.

[36] Maia D, de Carvalho AC, Horst MA, Carvalho AL, Scapulatempo-Neto C, Vettore AL (2015). Expression of miR-296-5p as predictive marker for radiotherapy resistance in early-stage laryngeal carcinoma. J Transl Med.

[37] McDermott N, Meunier A, Wong S, Buchete V, Marignol L (2017). Profiling of a panel of radioresistant prostate cancer cells identifies deregulation of key miRNAs. Clin Transl Radiat Oncol.

