# Supplementary material for: microRNA expression pattern as an ancillary prognostic signature for radiotherapy
Source: J Transl Med. 2018 Dec 5;16:341. doi: 10.1186/s12967-018-1711-4 (PMC6282371; doi:10.1186/s12967-018-1711-4)
Supplement: Supplementary file 1 — Additional file 1. Additional figures and tables. [file 12967_2018_1711_MOESM1_ESM.docx]

**Figure S1
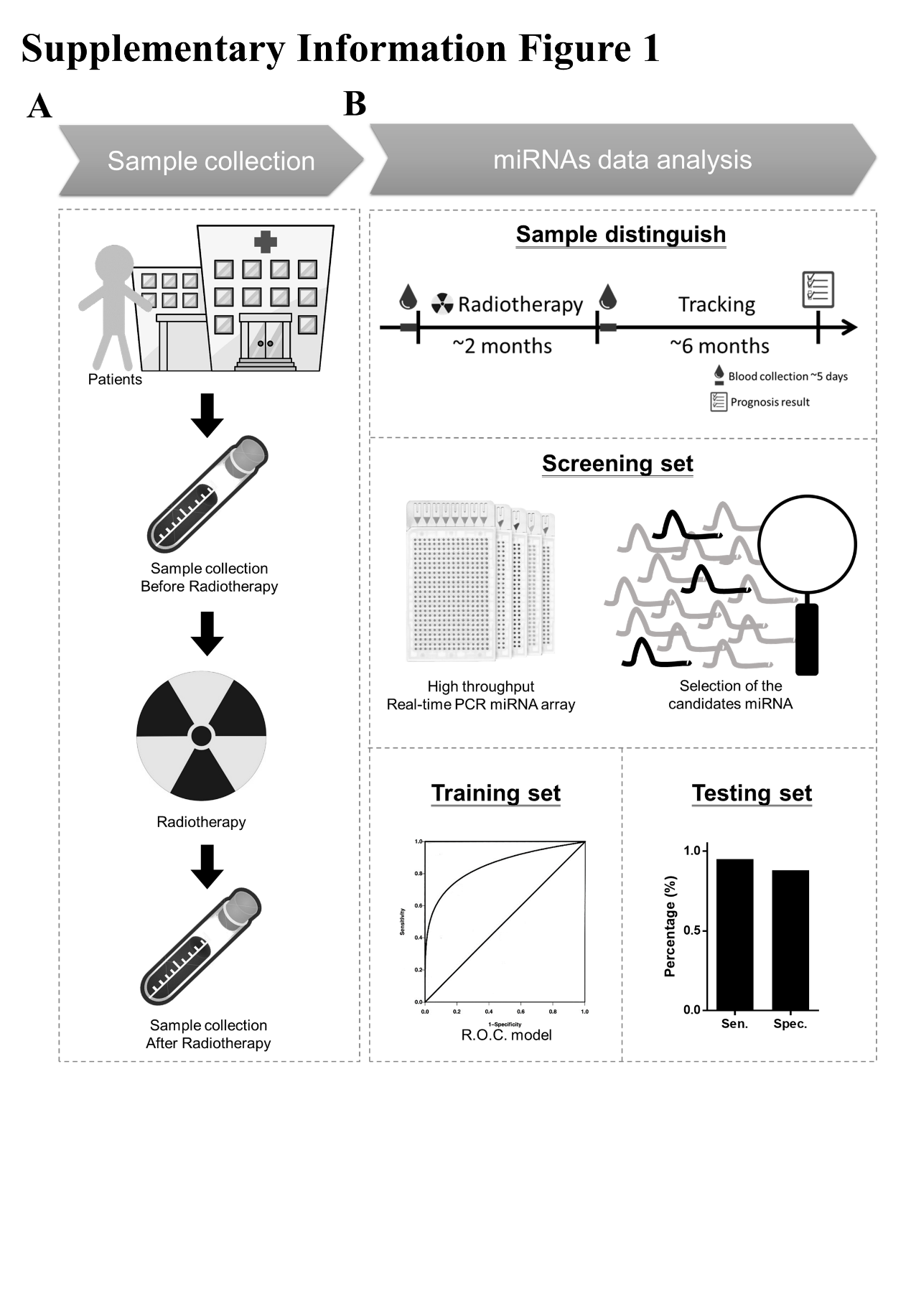
**

**Figure S1. The flow chart of the sample collection and experiment design.** (A) Plasma were extracted from patients and samples were collected before and after radiation therapy individually. (B) The illustration is displayed to identify and to verify candidate miRNAs for predicting the effect of Radiation treatment.

**Table S1
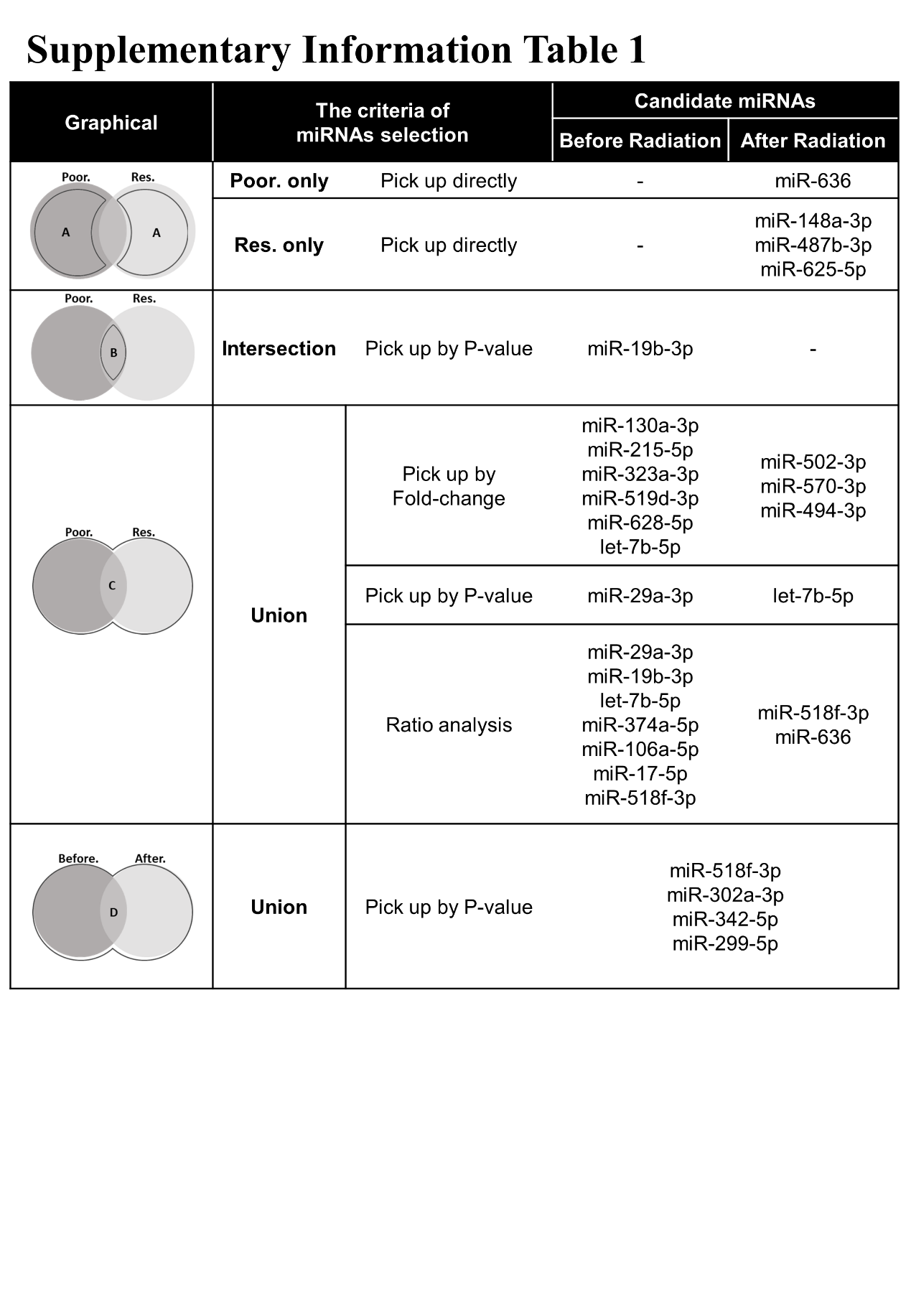
**

**Table S1. The Venn diagram and list of candidate miRNAs from screening set.** (A) the candidate miRNAs were selected by three ways. In A group, the miRNA only expressed in poor response or response samples. (B) the miRNA was selected by intersection and analyzed by student’s t test. (C) the miRNAs were filtered by union and the analyzed the ratio, fold change and student’s t test. (D) the miRNAs were filtered by union and the analyzed the student’s t test. Total 22 candidate miRNAs were selected for testing further to another group.

**Figure S2**

**Fig. S2. Significant changes in the miRNAs expression levels from same patients.** miRNA levels from the plasma of patients detected by qRT-PCR using RNU6 as a control. The Y axis presents the expression level (Log_10_ ^2-ΔCT^). Rad, radiation. Paired t-test: **P* value < 0.05; ***P* value < 0.01; ****P* value < 0.001

**Figure
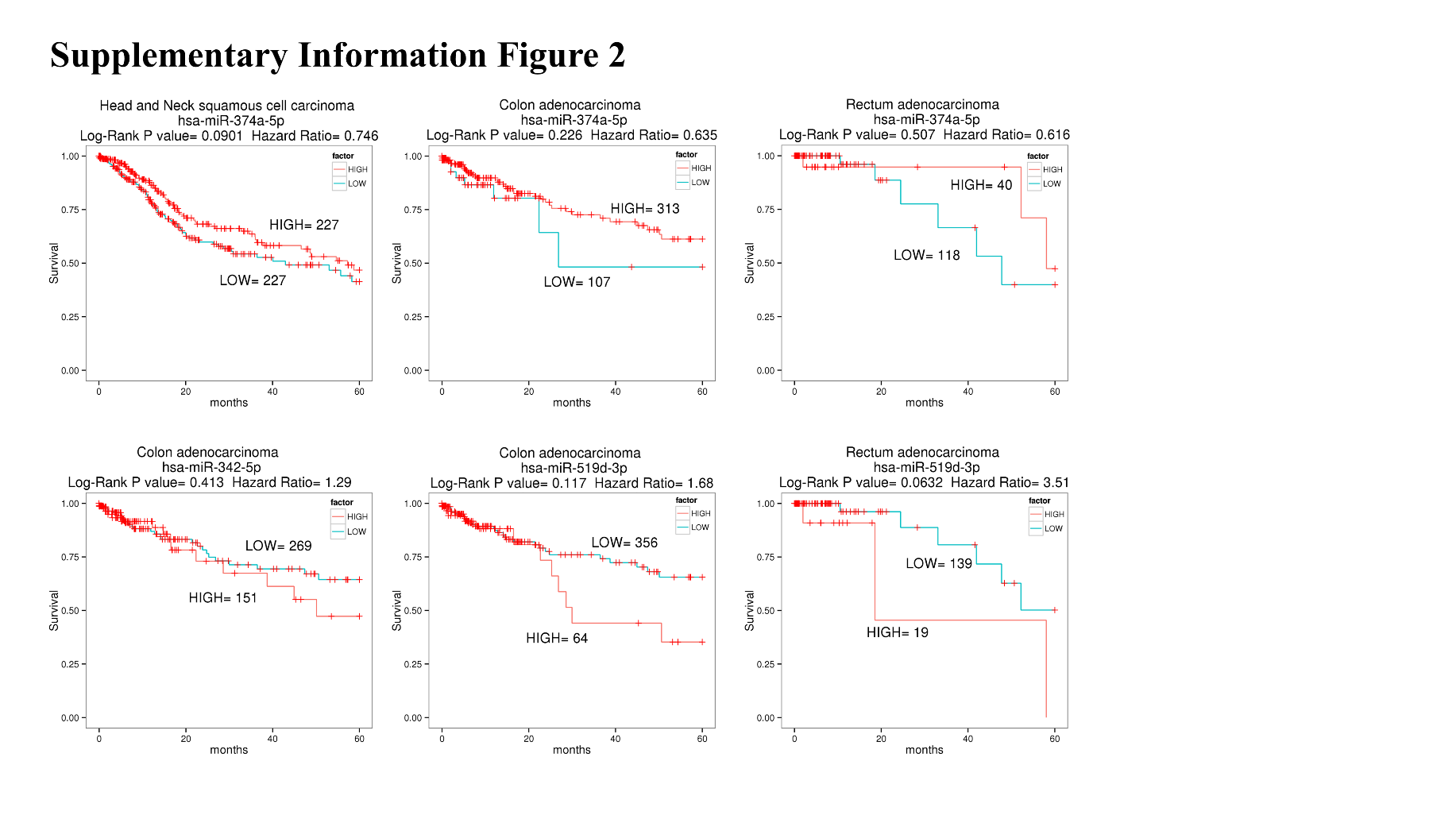
S3**

**Figure S3. The expression of pre-radiotherapy miRNA candidates in human cancer tissue is associated with survival.** The Kaplan-Meier survival curve of patients: low miRNA expression versus high miRNA expression. The statistical significance of the difference between the two groups was showed.

**
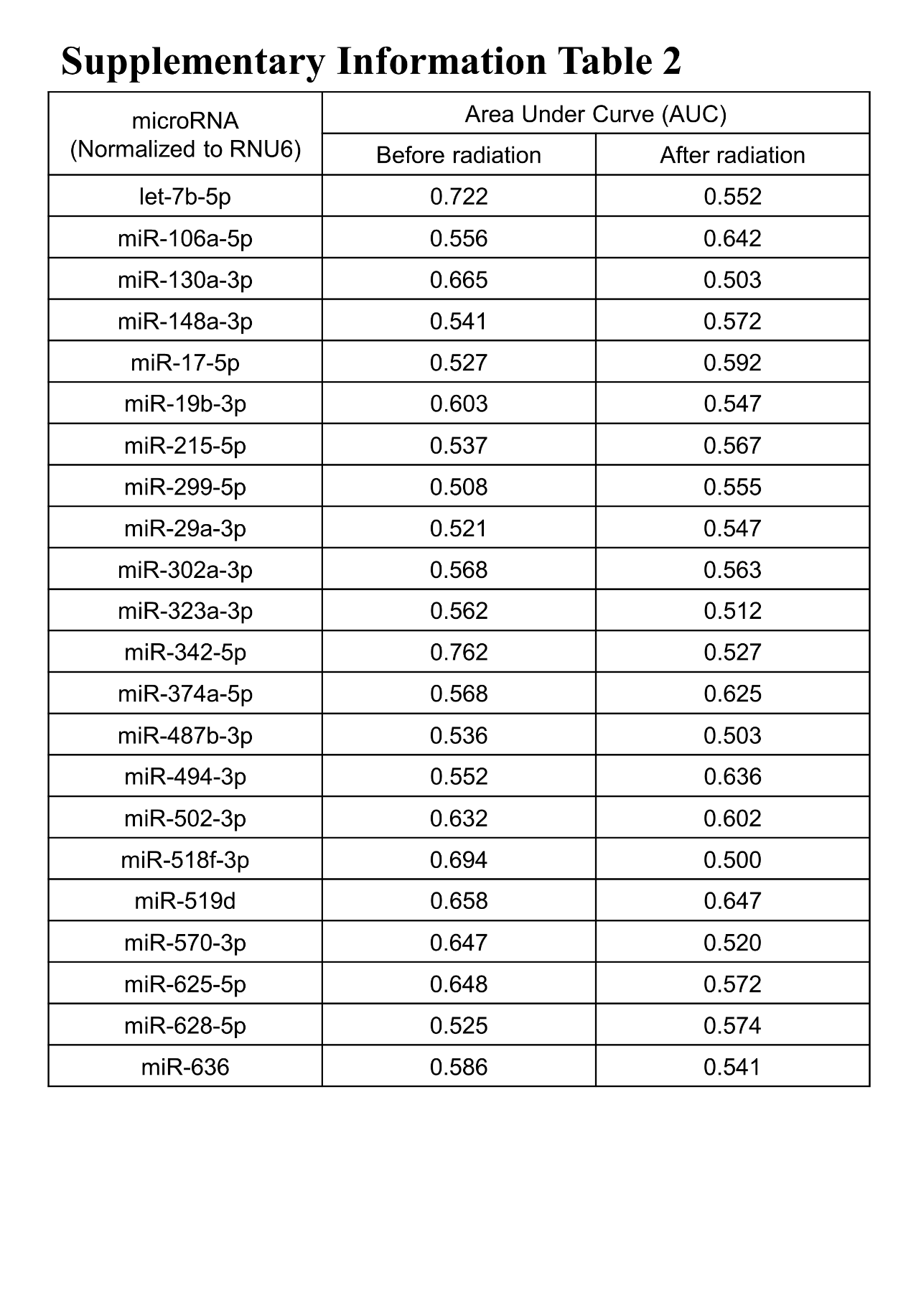
Table S2**

**Table S2. The AUC of candidate miRNAs from screening set.** The ROC analysis for the candidate miRNAs are shown to distinguish responsive or poor responsive patients of pre-radiotherapy or after radiation treatment.
